# Virtual Reality Therapy in Social Anxiety Disorder

**DOI:** 10.1007/s11920-020-01156-1

**Published:** 2020-05-13

**Authors:** Paul M. G. Emmelkamp, Katharina Meyerbröker, Nexhmedin Morina

**Affiliations:** 1 Department of Clinical Psychology, University of Amsterdam, Amsterdam, Netherlands; 2 Department of Clinical Psychology, Utrecht University, Utrecht, Netherlands; 3 Institute of Psychology, University of Münster, Münster, Germany

**Keywords:** Virtual reality exposure therapy, Social anxiety disorder, Speech anxiety, Assessment, Virtual social worlds

## Abstract

**Purpose of Review:**

This review provides an overview of current methods and important aspects to consider when applying virtual worlds in the treatment of social anxiety disorder (SAD).

**Recent Findings:**

Different aspects such as dialogs between avatars and patients have been investigated as well as virtual audiences, emotional facial expression, and verbal interaction with avatars. Results of these studies are promising. Few randomized controlled trials (RCTs) have investigated the efficacy of virtual reality exposure therapy (VRET) in SAD. Unfortunately, most RCTs into the efficacy of VRET in comparison with exposure in vivo in SAD have been conducted with a combination of cognitive interventions and VRET. No differences between these conditions were found, but the pure effect of VRET as a stand-alone treatment has only been investigated in one RCT, wherein VRET was not superior to exposure in vivo.

**Summary:**

Current research into different facets of SAD and VRET has produced promising results with respect to technological aspects. No differences in efficacy between cognitive behavior therapy and VRET were found, but there is a clear need for studies investigating the efficacy of VRET as a stand-alone treatment and the therapeutic processes involved before this therapy can be disseminated in routine clinical practice.

## Introduction

Social anxiety disorder (SAD) is characterized by an excessive fear of negative evaluation and rejection by other people and a consistent fear of embarrassment or humiliation [1]. The most commonly reported fear relates to public speaking or speaking up in a meeting, which can be referred to as “performance only” subtype of SAD. However, a substantial number of individuals with SAD suffer from this condition in most social and performance situations (“generalized” subtype of SAD). SAD is one of the most common mental disorders in the general population, with an estimated lifetime prevalence ranging from 2 to 13%, depending on the diagnostic threshold [2, 3]. According to the NICE guidelines [4], cognitive-behavioral therapy (CBT) and anti-depressant medication (selective serotonin reuptake inhibitors, SSRIs) are the treatment of choice for SAD. A central component of CBT is an exposure that involves confronting feared stimuli while eliminating safety behaviors so that patients learn that feared negative consequences are unlikely to occur. However, given the nature of the disorder, situations to practice in CBT are often scarce and difficult to reproduce. Virtual reality exposure therapy (VRET) has become an important therapeutic instrument to mimic social situations that are relevant within a therapeutic context and it has been shown to have the potential to elicit the social distress patients’ experience [4, 5]. However, research into the efficacy of VRET as a stand-alone treatment in SAD has been scarce and often results remain inconclusive [6, 7••, 8, 9•].

Although a number of virtual reality environments have been developed in recent years, research into SAD and VRET is still scarce. This might be due to the difficulty in developing virtual worlds that promote real-time human interaction.

In this review, we give an overview of current methods and important aspects to consider when applying virtual worlds in the treatment of SAD. Accordingly, we will review the most relevant technological aspects, which have been investigated, and discuss the potential of virtual reality as an assessment instrument and the efficacy and process variables of VRET relevant for therapeutic purposes. Finally, we will discuss future directions for SAD and virtual reality.

## Virtual Social Worlds

Several studies have investigated whether virtual social environments can be effectively manipulated for therapeutic purposes. Hartanto et al. [10] investigated two aspects of virtual social worlds: the social dialog situation and the dialog feedback responses. In the first study, 16 healthy participants were exposed to a neutral virtual world, a virtual blind date world, and a virtual job interview world. Results showed that exposure to the social worlds was associated with higher self-reported anxiety and heart rate. In the second study, the authors exposed 24 healthy participants to a virtual job interview scenario and systematically varied the ratio between negative and positive dialog feedback responses of the virtual character. Results revealed that positive dialog feedback was associated with significantly less self-reported anxiety, lower heart rate, and longer answers than negative dialog feedback. Similarly, Kishimoto et al. [11] instructed 26 individuals with SAD and 26 healthy controls to give two 3-min speeches and examined the impact of ambiguous and negative virtual social feedback. Compared with healthy controls, individuals with SAD reported higher levels of subjective anxiety and the difference was larger in the ambiguous condition than in the negative condition. Felnhofer et al. [12] too reported significantly higher levels of anxiety, co-presence, and immersion in participants with SAD than in healthy controls. Kim et al. [13] exposed 79 individuals with SAD and 51 healthy controls to impromptu speeches on self-related topics to a virtual audience and concluded that individuals with SAD demonstrate less eye gaze towards the audience than healthy controls. Lange and Pauli [14] explored avoidance behavior among individuals with high vs. low social anxiety (*n* = 50). The authors concluded that avoidance behavior when bypassing virtual humans with neutral and angry facial expressions is modulated by the emotional facial expressions of virtual bystanders and that social anxiety generally amplifies avoidance. Taken together, these studies suggest that virtual social environments can be successfully used for therapeutic purposes. In another study [15], the potential use of virtual immersive experiences in an omnidirectional video (or 360° video) was investigated for therapeutic purposes. A 360° video consists of a spherical recording of a real scenario. In this study, non-clinical participants delivered three speeches that were accompanied by a different type of reaction from the virtual audience. Exposure to the negative audience was associated with increases in skin conductance, heart rate variability, perceived anxiety, a higher ratio of silent parts in the speech, and lower social presence relative to exposure to the neutral audience [15].

Our research team [16] investigated in participants with high levels of social anxiety the use of verbal interactions between clients and virtual humans. We applied a virtual reality system specifically designed to expose clients with social anxiety complaints to anxiety-provoking social situations. Two sessions of virtual exposure involving several free speech dialogs with virtual humans while being monitored by a therapist were associated with significantly lower levels of social anxiety and higher self-efficacy 3 months after exposure. In the following study, we went a step further by developing a home-based VRET system for the treatment of SAD [17]. The so-called Memphis system includes 19 exposure scenarios and a virtual health agent that explains the use of the system and guides the patient to various steps of therapy. This system consists of (a) the virtual health agent application, (b) the VR system, and (c) the therapist application. The home-based program contains a laptop, an HMD, a heart rate sensor, a microphone, an Internet dongle, and a system manual operation handbook. Additionally, the therapist can set the treatment plan and monitor treatment progress remotely by using the therapist application, while data exchange is saved on a secure remote server. In another study [18], we set up a pilot study with patients with SAD who were scheduled to receive one introduction session, eight exposure sessions, and one relapse prevention session. Due to technical difficulties (such as unexpected software crashes or patients forgetting to charge batteries of the mouse or the wireless hear rate device), only one of the five patients staring treatment was able to successfully complete the whole treatment.

## Assessment

Can virtual social words be used for the clinical assessment of SAD? Powers et al. [19] were the first to demonstrate in a healthy sample that a virtual reality conversation task led to a similar increase in feelings of anxiety in participants as an in vivo conversation task. Recently, research has investigated whether VR can also be used to reliably and validly assess social anxiety and public speaking anxiety, but results are inconclusive. Kampmann et al. [20] investigated whether a virtual reality Behavioral Assessment Task (BAT) predicted social anxiety in daily life. The virtual reality BAT consisted of two virtual situations in which participants had to speak with virtual humans. Two situations were used in the virtual reality BAT: one representing the fear of small talk with unknown people and the other representing fear of speaking in public. The virtual situations used in the BAT consisted of (1) engaging in a conversation with a stranger at a bus stop and (2) attending a foreign language class in which the teacher asked the participant a number of questions. The dialogs were automated by means of speech detection technology [18]. Participants (healthy adults) rated their anxiety during the virtual reality BAT using the subjective unit of distress (SUD) scale from 0 (i.e., no distress) to 10 (unbearably upset). Participants were also instructed to give an impromptu 5-min speech (in vivo BAT) in front of a camera with the experimenter being present and to rate their SUDs. Analyses revealed that participants with high SUDs during the virtual reality BAT reported higher social anxiety on the daily event survey [21], which they completed daily at home for 1 week. The in vivo BAT did not prove to be a significant predictor of everyday social anxiety as assessed by the State Social Anxiety Questionnaire [22]; the virtual reality BAT did slightly better and approached statistical significance as a predictor for social anxiety. Although the results do not support the use of virtual reality BAT as an assessment tool for social anxiety, technical improvements in the near future including the ability to vary facial expressions might enhance the predictive validity of the virtual reality BAT.

Other studies have investigated potential physiological differences between subjects undergoing a virtual reality BAT or an in vivo BAT. In a study [23] with healthy subjects who had to give a presentation in front of a virtual audience and another group of subjects who had to give a presentation in front of a real audience, both groups showed significant increases in salivary cortisol and in cardiovascular activity. There were no differences in these measures between the virtual reality presentation group and the in vivo presentation group. Owens and Beidel [6] found that having to give a speech for a virtual audience led to an increase in physiological arousal (electrodermal activity, respiratory sinus arrhythmia, and heart rate)—despite the only limited presence in the VR environment—but the increase in arousal was slightly less than having to give a speech to a real audience. This applied both to normal participants and individuals with SAD. One study [24] suggests that analyzing eye movements (fixation duration of faces) during virtual reality BAT using social situations may be a better predictor for distinguishing low vs. high social anxiety levels than electrodermal activity. Finally, in a study with subjects with public speaking anxiety [25] who were wearing a wrist-based sensor during a virtual reality BAT, the investigators were able to predict a four-class anxiety level with an accuracy of up to 86%. This was based on a combination of blood volume pressure (BVP), galvanic skin response (GSR), and skin temperature.

Although most studies so far have been conducted with adults, at least one study [26] found that virtual reality BAT also leads to an increase in anxiety in adolescents between the ages 13 and 18. Socially anxious adolescents reacted with higher anxiety in a virtual party and in a virtual speech presentation as compared with neutral virtual environments.

## Effects of Virtual Reality Exposure Therapy

### Randomized Controlled Trials investigating VRET

But what do we know about the effectiveness of virtual reality exposure therapy in SAD? Is virtual reality exposure therapy (VRET) ready for dissemination in the routine treatment of patients with SAD? A number of studies have investigated the efficacy of virtual reality exposure therapy in patients with SAD or public speaking anxiety, but we will limit our review to controlled studies, which are considered more reliable because they account for the impact of time on symptoms.

The first study with patients with SAD in which VRET was compared with a control condition [27] was not an RCT: Patients with SAD were divided over two groups and matched based on the following variables: gender, age, duration, severity of social phobia, ability to use computers, or virtual reality software. Two treatments were compared: (a) 12 sessions of VRET, consisting of exposure to five virtual environments that cover diverse social situations and (b) 12 sessions of group cognitive behavior therapy. VRET was as effective as group cognitive behavior therapy.

More recently four RCTs have investigated VRET in social anxious patients, including speech anxiety. Bouchard et al. [28] compared two variants of CBT plus exposure with waiting list control: (a) CBT plus exposure in vivo and (b) CBT plus exposure in virtuo. Both variants of CBT were clearly more effective than the control condition and no differences were found in the effects of CBT plus exposure in vivo and CBT plus exposure in virtuo. Results are difficult to interpret, however, given that the exposure variants were mixed with other CBT exercises.

In an RCT by Anderson et al. [29], patients were randomly assigned to 8 weeks of (a) VRET, (b) exposure group therapy, and (c) waiting list control. Both treatments involved cognitive components addressing self-focused attention, negative perception of self and others, perception of negative emotion regulation, ruminations, and unrealistic goal settings in social situations. A substantial number of participants had a fear of public speaking as their main complaint. Results were re-assessed 4 to 6 years after treatment and the majority of patients reported significant improvements [30]. Of note, results at follow-up were limited to 28 of the original 65 patients who completed treatment in the RCT.

Bouchard et al. [31] compared three conditions: CBT plus VRET, CBT plus in vivo exposure, and waiting list. Both active treatments were more effective than waiting list and CBT plus VRET was more effective than CBT plus in vivo exposure. Results were maintained up to a 6-month follow-up. In this study, the therapist was in the same room as the patient and there was intensive interaction between patient and therapist during the virtual exposure. Therapists used this interaction to discuss the exposure experiences of the patient during VRET, which may have confounded the results [32]. Again, given the combination of cognitive restructuring and VRET, the effects of VRET are difficult to evaluate.

The RCT by Kampmann et al. [7••] is the only study so far with patients with generalized SAD where pure VRET without any cognitive intervention was investigated. Pure individual VRET was compared with individual exposure in vivo without any cognitive intervention and a waiting list control group. This study was the first attempt to develop and apply a variety of complex virtual social interactions: the virtual situations consisted of giving a talk in front of an audience of people, who asked questions: buying and returning clothes; talking to a stranger; attending a job interview; dining in a restaurant; being interviewed by a journalist; and having a blind date. Semi-structured dialogs were controlled by the therapist, who was in a separate room. The therapist could vary dialog style, gender of avatar, avatar’s gestures, number of avatars present, and dialog topic’s degree of personal relevance. Exposure in vivo consisted of exposure exercises that could be implemented in the office of the therapist, or in supermarkets, cafés, shops, or subway stations in the neighborhood of the office. Both active treatments were more effective than the waiting list control group on social anxiety symptoms, BAT, stress, and avoidant personality disorder related beliefs. However, in vivo exposure was more effective than VRET in reducing social anxiety and avoidant personality disorder-related beliefs at a 3-month follow-up. Thus, although VRET as a stand-alone therapy containing extensive verbal interaction without any cognitive restructuring was effective in reducing complaints of generalized SAD, it was still less effective than exposure in vivo.

### Meta-analyses

A number of meta-analyses have been published. Kampmann et al. [5] reported that when VRET for SAD was compared with passive control conditions at post-assessment, the effect size was large (Hedges’ *g* = 0.82); when compared with active control conditions, the effect size was not significant (g = − 0.24). This was confirmed in a meta-analysis of Chesham et al. [33]. Wechsler et al. [34] published a meta-analysis on RCTs specifically comparing the effectiveness of VRET to exposure in vivo in anxiety disorders including RCTs discussed above in SAD [7••, 29, 31]. For all anxiety disorders together, the comparison of VRET to exposure in vivo revealed a small non-significant effect size (*g* = 0.20) in favor of exposure in vivo, corroborating results of Carl et al. [35]. One meta-analysis [9•] found support for the generalization of the effects of VRET to real life, but this review was limited to studies with specific phobias.

The finding that VRET appears to be less effective than exposure in vivo only in patients with SAD may be related to the fact that it is still far more difficult to create realistic virtual social environments for use in VRET in patients with SAD in contrast to VR worlds for patients with specific phobias such as fear of heights, fear of animals, or fear of flying and VR worlds for patients with agoraphobia. On the other hand, comorbidity with other anxiety disorders and depression [36] and avoidant personality disorder [37] is much higher in patients with SAD than in patients with specific phobias. Whether this comorbidity is related to the outcome of VRET in SAD deserves to be studied.

### Pharmacological Augmentation of VRET

In recent years, different pharmacological augmentation strategies for exposure-based treatments have been investigated in anxiety disorders [38], including yohimbine hydrochloride and D-cycloserine (DCS), which have both been investigated in SAD.

While the effects of yohimbine have been investigated more extensively in other anxiety disorders and also in VRET with specific phobias [39], research into the effects of yohimbine in the treatment of SAD is scarce. Only one RCT has been done in individuals with SAD [40], but this has not been studied yet in VRET. DCS research findings have been more promising than yohimbine outcomes [41, 42]. Although it is argued [43] that when DCS is administered at the right time, in the exact dosage and with the right number of exposure sessions, DCS has a positive impact as an augmentation strategy; there is a clear need of investigating this in VRET in patients with SAD before this can be implemented in clinical practice.

### Attrition and Deterioration

One of the potential advantages of virtual reality exposure therapy (VRET) for SAD is that there may be less attrition from therapy when exposure to social situations is virtual rather than in real life as is the case in exposure in vivo [44]. Attrition means discontinuation of therapy before the therapy has been completed. In a recent meta-analysis of attrition in VRET for anxiety disorders [45], three RCTs were included that compared VRET and exposure in vivo in SAD [7••, 29, 31]. The authors concluded that the results of these trials suggest that attrition rates are not lower in VRET than exposure in vivo.

But what about the negative effects of VR treatment? In a recent study [46•], the deterioration rates of VR were investigated in retrieved datasets from 15 published randomized controlled trials for anxiety disorders including 4 RCTs with SAD. Deterioration was established with the Reliable Change Index [47]. Overall results showed that deterioration rates for VR therapy were comparable with other therapeutic approaches and the deterioration rate was less for patients treated with VR therapy as compared with patients in waiting list control groups. However, results were not analyzed for each specific anxiety disorder.

## Process-Related Variables

### Therapeutic Alliance in VRET

Very few studies have investigated the processes involved in VRET. One of the most investigated processes in psychotherapy in general is therapeutic alliance [48], but this has hardly been investigated in VRET. Meyerbröker and Emmelkamp [49, 50] suggested that the working alliance may be impaired in VRET due to the fact that patients wear head-mounted displays, thus preventing eye contact. In the abovementioned trial by Anderson et al. [29], no difference was found between the level of the working alliance in the VRET and exposure group therapy [51]. Based on the same data set, Draheim and Anderson [52] found no mediation of the working alliance on the relation between outcome expectancy and improvement. However, as discussed above, both treatments consisted of a combination of cognitive restructuring and exposure, which renders results difficult to interpret.

### Attention Bias

According to cognitive models of SAD, patients with SAD are prone to biases in information processing. As a result, attention bias modification has been developed in order to modify the attention bias and thereby reduce social anxiety. Attention bias modification has been criticized [53] and recent meta-analyses of studies into the effects of attention bias modification in SAD found indeed disappointing results [5, 54]. As noted by Pelissolo et al. [55], the effect size for attention bias modification reported is smaller than standard placebo effect sizes in RCTs examining the treatment of SAD. A recent study of Ma et al. [56] employed virtual reality 3-D facial expression as stimuli in a dot-probe attentional bias modification task, but virtual reality-based attentional bias modification did not change the attentional bias and most participants did not clinically improve.

Benbow and Anderson [57] reported that in the sample of patients of the Anderson et al. study [29], self-report of probability and cost biases following social events was improved after treatment with no differences between virtual reality exposure and exposure group therapy. Although the probability and costs of negative outcomes in social situations are related to social anxiety according to Clark and Wells [58], the positive results of the Benbow and Anderson study [57] can probably be attributed to the cognitive part of the treatment rather than to the exposure component. Finally, in a study by Kampmann et al. [59], change in attention bias after treatment with VRET did not significantly differ from the change in the waiting list condition.

## Future Directions

Given the social nature of SAD, future research needs to develop extensive and flexible dialogs to be used in VRET and examine how they can further improve the usability of VRET. Such verbal interaction should allow for more realistic and unpredictable social interaction and include individualized responses. At the same time, more virtual situations that enable verbal interactions need to be developed. Both stimuli that trigger social anxiety within a certain social situation as well as the incorporated verbal interactions need to be flexible and allow for adjustment to the individual’s needs by adding or removing social situations. Additionally, technological developments enable the implementation of facial expressions of virtual humans, which in turn might improve the efficacy of VRET.

In a recent study [60], patients with generalized SAD were treated with virtual reality-based cognitive behavioral therapy, which has been found to be effective in patients with psychotic disorders [61]. During VR exercises, patients tested whether their beliefs were rational and exposure to virtual social situations was practiced and therapists gave feedback on cognition and behavior. In addition, cognitive restructuring was applied by the therapist. In this pilot study, this form of virtual reality-based cognitive behavioral therapy proved to be effective in reducing anxiety and depressed mood, but the study lacked a control condition. Further studies are needed to investigate whether this treatment format is more effective than VRET as a stand-alone treatment in patients with generalized SAD.

Most studies into treatment for SAD employed virtual reality systems with immersive headsets. A few studies used computer-generated 3-D scenes, but these studies were uncontrolled [62, 63]. For the technical differences between immersive virtual reality and computer-generated 3-D scenes, see Cipresso et al. [64]. There is a clear need for comparing the effectiveness of VRET using immersive headsets versus VRET using computer-generated 3-D scenes in patients with SAD. One of the issues to be investigated is whether the presence in the VR world defined as the feeling of “being there” is comparable in VRET using headsets versus VRET using 3-D images. If VRET using 3-D scenes is equally effective as VRET using headsets, it is likely that VRET using 3-D scenes will be more used by clinicians since it is easier to use in the office.

Another aspect remaining to be investigated is the use of developing virtual social environments, which ideally can be made interactive, versus the use of real-life filmed virtual environments, where individuals usually cannot interact with virtual avatars. The former is much more capable of immersing a person into an interactive environment, while the latter seems to be much more realistic visually, but usually do not provoke the same sense of being in a situation.

Until a few years ago, VR hardware was very expensive and needed a very expensive computer to run the program. The technological developments and off-the-shelf VR platforms currently available will help to enable the widespread application of VR. In a recent RCT [65], the authors investigated whether cheap consumer VR hardware and software can be used to conduct VRET for public speaking anxiety. Half of the participants with public speaking anxiety were treated with one 3-h VRET session combined with cognitive restructuring at the therapist office followed by a 4-week program consisting of VRET exercises at home and twice a week in vivo public speaking exposure exercises. The other half of the participants received—after a 5-week waiting period—the same treatment but fully self-led at home. Both active treatments led to large decreases in self-reported public speaking anxiety and improvements were maintained up to 12 months following treatment. Whether this self-led intervention can be easily applied in clinical practice remains to be further investigated. A limitation of this trial was that not all participants were formally diagnosed with SAD.

Nearly all studies investigating the effects of VRET in SAD involved adults. In a recent study [66], the effects of VRET were investigated in 27 adolescents between 13 and 16 years old with fear of public speaking. The VR treatment protocol was adapted from Lindner et al. [65] and consisted of one 90-min training session consisting of seven speech tasks in a virtual classroom with increasing difficulty. Although the treatment led to a substantial reduction in public speaking anxiety, the lack of a control group renders results difficult to interpret. Future studies need to compare VRET with evidence-based treatment for social anxiety in youth [67, 68]. There is also a clear need for studies investigating the effects of virtual reality in the elderly with SAD [69].

## Conclusions

Current research into different facets of SAD and VRET has produced promising results. Different aspects such as dialogs between avatars and patients have been investigated as well as virtual audiences, emotional facial expression, and verbal interaction with avatars. The development of technology is rather fast, but not yet stable enough to include all these components into VR worlds for clinical treatment. Generally, it can be concluded, that the more interactive a virtual environment is, the more immersive it is perceived.

Unfortunately, most RCTs into the efficacy of VRET in comparison with exposure in vivo in SAD have been conducted with a combination of cognitive interventions and VRET. No differences between these conditions were found, but the pure effect of VRET as a stand-alone treatment has only been investigated in one RCT [7••], wherein VRET was not superior to exposure in vivo. There is a clear need for further studies into the effectiveness of virtual reality treatment in patients with SAD and in the processes involved before this therapy can be disseminated in routine clinical practice.
